# Skull Bone Marrow as a Cranial Neuroimmune Reservoir in Alzheimer’s Disease: Amyloid, Osteoimmune Remodeling, and Translational Imaging Evidence

**DOI:** 10.3390/cells15191738

**Published:** 2026-09-23

**Authors:** James Chmiel, Jerzy Leszek

**Affiliations:** 1 Faculty of Physical Culture and Health, Institute of Physical Culture Sciences, University of Szczecin, Al. Piastów 40B Block 6, 71-065 Szczecin, Poland; 2 Department of Psychiatry, Wroclaw Medical University, 50-367 Wroclaw, Poland

**Keywords:** Alzheimer’s disease, skull bone marrow, amyloid-β, meningeal lymphatics, neuroinflammation, osteoimmunology, cerebrospinal fluid, cranial immune reservoir

## Abstract

Alzheimer’s disease (AD) has traditionally been conceptualized as a disorder driven primarily by cerebral amyloid-β accumulation, tau pathology, synaptic failure, vascular dysfunction, and chronic neuroinflammation. However, emerging evidence suggests that AD pathobiology may also involve extracerebral cranial compartments positioned at the interface between the brain, meninges, cerebrospinal fluid, and skull bone marrow. Recent anatomical and experimental studies have identified direct vascular and osseous channels connecting skull bone marrow with the dura mater, allowing bidirectional exchange of immune cells, soluble mediators, and cerebrospinal fluid-derived signals. These findings support the concept that skull bone marrow functions as a specialized cranial immune reservoir capable of sensing central nervous system-derived molecular cues and supplying myeloid and lymphoid cells to meningeal and brain-border compartments. In AD models and human imaging studies, amyloid-β-related signals have been detected in skull marrow, where they may promote IL-6-dependent B lymphopoiesis, expansion of age-associated B cells, microglial activation, and amplification of cerebral amyloid pathology. In parallel, AD-related amyloid precursor protein and amyloid-β signaling may remodel the skull marrow niche through osteoblast dysfunction, altered osteoclast activity, vascular channel expansion, marrow adiposity, and inflammatory reprogramming. Human PET and MRI studies indicate that skull marrow-associated inflammatory, amyloid-related, and cerebrospinal-fluid drainage signals can be detected in vivo; however, these observations remain indirect, method-dependent, and insufficiently validated for diagnostic or prognostic use. Importantly, much of the anatomical foundation for skull marrow–meninges–brain communication derives from non-AD studies of CNS-border physiology, stroke, meningitis, spinal cord injury, and infection, whereas the strongest AD-specific mechanistic evidence is currently derived from transgenic mouse models. Accordingly, we frame the skull marrow–meninges–brain axis as a testable conceptual model rather than an established causal pathway in human AD. This review integrates anatomical, immunological, osteoimmune, and imaging evidence while explicitly distinguishing AD-specific findings from evidence extrapolated from other neurological or inflammatory contexts. Determining whether skull marrow alterations represent a cause, consequence, compensatory response, or amplifier of AD pathology will require longitudinal, biomarker-defined human studies and further mechanistic validation. The review also compares the principal in vivo strategies used to distinguish skull-marrow-derived immune cells from circulating leukocytes and examines ischemic stroke and brain tumors as comparator CNS conditions. These disease models demonstrate that cranial marrow responses can be rapid, spatially organized, and either protective or pathogenic depending on cellular phenotype and disease context, reinforcing the interpretation of skull marrow as a general CNS-border immune organ rather than an AD-specific compartment.

## 1. Anatomical Basis

The anatomical rationale for considering skull bone marrow as a cranial neuroimmune reservoir arises from a major revision of the classical view of the central nervous system (CNS) as an immunologically isolated compartment. The brain is enclosed by the skull and meninges, but this interface is not a passive mechanical barrier. Rather, the dura mater, arachnoid mater, pia mater, cerebrospinal fluid (CSF), dural venous sinuses, meningeal lymphatics, and calvarial bone marrow form a highly specialized border system that permits immune surveillance, solute exchange, and regionally organized communication between the CNS and adjacent extracerebral tissues. The outer meningeal layer, the dura mater, is directly apposed to the inner skull surface and contains blood vessels, venous sinuses, lymphatic vessels, resident and trafficking immune cells, and anatomical continuities with skull marrow spaces, making it the central anatomical bridge between brain-derived signals and cranial hematopoiesis.

An important distinction is required when interpreting this anatomical literature. The existence of direct skull marrow–meninges channels, meningeal lymphatic drainage, CSF-to-skull molecular exchange, and local cranial immune-cell trafficking is increasingly well supported, but these observations do not by themselves establish a pathogenic skull marrow–meninges–brain axis in Alzheimer’s disease (AD). Several foundational studies discussed below were conducted in healthy animals or in models of stroke, aseptic meningitis, bacterial meningitis, spinal cord injury, or other inflammatory conditions. We therefore use these studies to establish anatomical and physiological plausibility, not as direct evidence that the same processes are quantitatively or functionally important in human AD. AD-specific conclusions are distinguished from this broader anatomical framework throughout the review.

A first conceptual shift came from the rediscovery of meningeal lymphatic vessels. Louveau et al. demonstrated functional lymphatic vessels lining the dural sinuses that express lymphatic endothelial markers and transport fluid and immune cells from the CSF toward deep cervical lymph nodes [1]. In parallel, Aspelund et al. showed that dural lymphatic vessels drain CSF and brain interstitial fluid, thereby providing an anatomical pathway through which CNS-derived macromolecules may exit toward peripheral lymphatic compartments [2]. Subsequent work refined this model by showing that basal meningeal lymphatic vessels at the skull base have specialized morphology and play a particularly important role in CSF drainage [3]. These findings are important for AD, because impaired meningeal lymphatic drainage has been shown experimentally to exacerbate amyloid-β deposition, microglial activation, and age-related cognitive decline, while enhancement of meningeal lymphatic function can improve responses to anti-Aβ immunotherapy.

A second anatomical shift came from the discovery that the skull bone marrow is directly connected to the meninges through microscopic vascular and osseous channels. Hérisson et al. identified direct vascular channels crossing the inner skull cortex and connecting skull bone marrow with the dura and brain surface. In mouse models of stroke and aseptic meningitis, skull marrow-derived neutrophils were more likely than tibial marrow-derived neutrophils to migrate toward adjacent inflamed brain regions, indicating that cranial marrow can provide a local immune supply to the CNS surface [4]. Cai et al., using whole-body clearing and panoptic imaging, independently visualized short vascular connections between skull marrow and the meninges, which became populated by immune cells after CNS injury [5]. Together, these studies established that the calvarial marrow is not merely a distant hematopoietic organ located near the brain; it is anatomically positioned to communicate locally with the meningeal compartment.

This skull–meninges communication is functionally relevant for both myeloid and lymphoid immune lineages. Cugurra et al. showed that skull and vertebral bone marrow niches act as myeloid reservoirs for the meninges and CNS, supporting the concept that CNS-associated bone marrow compartments can supply immune cells to border tissues under homeostatic and inflammatory conditions [6]. Brioschi et al. extended this concept to adaptive immunity by demonstrating that the meninges contain heterogeneous B-cell populations, including cells derived locally from skull bone marrow, suggesting the presence of a lymphopoietic niche at CNS borders [7]. These findings are highly relevant to AD because emerging evidence implicates both myeloid cells and B-cell subsets in amyloid-associated neuroinflammation, and because skull-derived immune cells may reach the meninges and, under pathological conditions, the brain parenchyma.

Importantly, communication across the skull–meninges interface is not unidirectional. Several studies indicate that CSF-borne signals can access skull bone marrow and modulate local hematopoiesis. Mazzitelli et al. reported that CSF reaches skull marrow niches through dura–skull channels and that CSF proteins signal to multiple cell types within the marrow microenvironment; after spinal cord injury, CSF-borne cues promoted myelopoiesis and egress of myeloid cells toward the meninges [8]. Pulous et al. similarly showed that CSF can exit into skull bone marrow through perivascular routes and numerous skull channels; in bacterial meningitis, this route enabled microbial and inflammatory signals to reach cranial hematopoietic niches and initiate skull marrow responses earlier than remote long-bone marrow [9]. Thus, the skull marrow may function as a sensor of CNS-derived molecular information, rather than solely as a supplier of immune cells.

Human observations support the translational plausibility of skull marrow–CNS exchange. Intrathecal MRI demonstrates movement of CSF tracer into skull marrow, systematic synthesis confirms cross-compartment passage of cells and solutes, and multimodal studies indicate that skull marrow has measurable drainage and inflammatory signatures as well as molecular features distinct from remote marrow [10,11,12,13]. These human and translational findings are examined in detail in Section 6.

Together, these studies establish anatomical and physiological communication among skull bone marrow, the meninges, CSF, and CNS-border compartments through vascular channels, molecular exchange, and local immune-cell trafficking. However, much of this evidence derives from non-AD experimental settings and therefore establishes biological plausibility rather than a demonstrated causal skull marrow–meninges–brain pathway in human AD. As summarized in Figure 1, the foundational anatomical and physiological framework gives rise to three principal evidence domains = meningeal lymphatic and CSF drainage, skull–meninges channels and local immune-cell trafficking, and CSF-to-skull marrow communication—which together support subsequent AD-specific experimental observations and more indirect human translational findings. The figure therefore summarizes the evidential structure of the field and the current cautious interpretation of its relevance to human AD.

A simplified cross-sectional cartoon of this anatomy is provided in Figure 2, emphasizing the close apposition of calvarial marrow and dura, vascular/osseous skull–meninges channels, meningeal vascular and lymphatic structures, the subarachnoid CSF compartment, and the bidirectional concept of immune-cell egress and CNS-derived molecular signaling. Anatomical and physiological evidence supporting communication between skull bone marrow, the meninges, cerebrospinal fluid, and the brain is presented in Table 1.

## 2. Review Approach, Literature Search, and Evidence Classification

This article was designed as a structured narrative review aimed at integrating the emerging literature on skull bone marrow, skull–meninges communication, cranial hematopoiesis, and their potential relevance to AD. Because the available evidence spans heterogeneous experimental paradigms, including human imaging and tissue studies, transgenic AD models, acute neurological injury models, infection models, and studies of general CNS-border biology, the objective was not to perform a quantitative meta-analysis but to provide a transparent, evidence-stratified synthesis of this developing field. The literature-search and study-selection procedures were specified to improve reproducibility while retaining the broader scope appropriate for a structured narrative review.

A literature search was conducted in PubMed/MEDLINE, Scopus, and Web of Science from database inception to 11 June 2026. The search strategy combined terms describing the cranial marrow compartment and CNS-border anatomy with terms related to AD, amyloid pathology, neuroinflammation, hematopoiesis, and osteoimmune remodeling. Core concepts included ‘skull bone marrow,’ ‘calvarial bone marrow,’ ‘cranial bone marrow,’ ‘skull marrow,’ ‘skull-meninges,’ ‘dura-skull channels,’ ‘cranial hematopoiesis,’ ‘meningeal lymphatics,’ ‘cerebrospinal fluid,’ ‘Alzheimer’s disease,’ ‘amyloid,’ ‘amyloid-beta,’ ‘APP,’ ‘neuroinflammation,’ ‘osteoblast,’ and ‘osteoimmune,’ together with database-specific spelling and indexing variants. The complete database-specific search strategies, including exact Boolean search strings, field specifications, database-specific syntax, and dates of the final searches, are provided in Supplementary Table S1. No publication-date restriction was applied. Only English-language publications were considered. Reference lists of eligible original studies and relevant reviews were additionally screened to identify potentially relevant publications not retrieved through the database searches. For the present revision, a focused literature update was conducted through 18 September 2026 to address two editor-requested domains not emphasized in the original AD-centered synthesis: (i) in vivo approaches for establishing the contribution and origin of skull-bone-marrow-derived immune cells, and (ii) skull marrow pathophysiology in ischemic stroke and primary brain tumors. The update combined targeted PubMed/MEDLINE searches with backward and forward citation screening of key primary studies and recent methodological syntheses.

The results of search blocks A and B were combined for each database. The revision-focused supplementary search was used specifically to identify methodological and comparator-disease literature requested during peer review. Reference lists of eligible original studies and relevant reviews were additionally screened to identify potentially relevant publications not captured by the electronic searches.

Duplicate records retrieved across databases were removed before screening. Retrieved records were assessed for relevance in two stages. First, titles and abstracts were examined to identify studies potentially addressing the predefined domains of interest. Potentially relevant articles were subsequently evaluated in full text to determine whether they provided evidence relevant to the skull marrow–meninges–brain framework. Eligibility was assessed independently by two authors, and disagreements regarding study inclusion or evidential classification were resolved through discussion and consensus. Studies were retained when they contributed evidence concerning at least one of the following domains: (1) anatomical or functional communication between skull bone marrow, dura mater, meninges, CSF, and the CNS; (2) skull or bone-marrow immune-cell production and trafficking relevant to CNS biology; (3) AD-, APP-, or Aβ-associated changes in skull marrow, bone, or cranial hematopoiesis; (4) human imaging or histological evidence relevant to the skull marrow–meninges interface; or (5) experimental interventions capable of modifying components of this system. Studies addressing peripheral bone marrow without a clear mechanistic or anatomical relationship to the cranial compartment were not considered direct evidence for the skull marrow–meninges–brain hypothesis.

Following study selection, evidence was classified according to its proximity to human AD and the disease context in which the relevant observation was demonstrated. Four categories were applied throughout the review: Level I, human AD-specific evidence, including studies of patients with clinically or biomarker-defined AD; Level II, AD-specific experimental evidence, including transgenic or experimentally induced AD-related models; Level III, human non-AD anatomical or translational evidence, including studies demonstrating skull–meninges, CSF, or marrow communication in populations not selected for AD; and Level IV, non-AD experimental or general CNS-border evidence, including studies of stroke, meningitis, spinal cord injury, infection, acute inflammation, or normal CNS-border physiology. Therapeutic concepts without direct testing in AD were considered hypothesis-generating rather than evidence of clinical efficacy.

This hierarchy was used to prevent anatomical plausibility demonstrated in non-AD systems from being interpreted as evidence of AD-specific causality. Accordingly, observations from Levels III and IV were used primarily to establish biological feasibility and potential mechanisms, whereas conclusions concerning AD pathogenesis were based preferentially on Levels I and II. No formal causal inference was made from the presence of skull–meninges anatomical communication alone.

Importantly, these categories describe evidential context and proximity to human AD rather than methodological quality or risk of bias. A Level I study is therefore not automatically methodologically stronger than a Level II study; rather, it provides evidence more directly situated within human AD. Study-specific methodological limitations were considered separately in the evidence tables and narrative synthesis.

Because this was a structured narrative review rather than a systematic review designed to estimate pooled effects, study inclusion was guided by mechanistic and translational relevance rather than by a narrowly defined intervention–comparator–outcome framework. No quantitative meta-analysis was planned because of substantial heterogeneity in populations, disease models, imaging methods, experimental interventions, biological compartments, and reported outcomes. The search procedure was nevertheless documented in sufficient detail to allow reproduction of the literature-identification process and to clarify how AD-specific evidence was distinguished from broader CNS-border evidence.

The framework developed in this review should not be interpreted as demonstrating that skull bone marrow is a necessary or sufficient driver of AD, that skull–meninges communication is specific to AD, or that anatomical connectivity implies disease causality. Likewise, detection of a skull-associated PET or MRI signal should not be equated with validation of a diagnostic or prognostic biomarker, and experimental modifiability of a pathway should not be equated with therapeutic efficacy. The purpose of the synthesis is instead to identify convergent observations, distinguish their evidential contexts, and formulate testable hypotheses for future experimental and longitudinal human studies.

## 3. Skull Bone Marrow as a Cranial Immune Reservoir

Beyond the anatomical connectivity described in Section 1, experimental studies indicate that skull and vertebral bone marrow can function as local sensor–effector compartments at CNS borders. Cranial marrow can supply myeloid and lymphoid populations to the meninges and CNS-associated tissues and can alter hematopoietic output in response to CNS-derived signals [6,7,8,9]. Although these foundational observations derive largely from non-AD models, they establish the functional capacity required for a local cranial immune reservoir.

AD-specific studies indicate that this capacity can become engaged during amyloid-related pathology. Aβ-associated changes in marrow hematopoiesis and IL-6-dependent B-cell expansion are considered in Section 4, whereas APP-associated osteoblastic remodeling, vascular-channel changes, and myeloid trafficking are examined in Section 5 [14,15]. Additional experiments show that skull-derived immune cells can enter amyloid-containing brain environments under selected conditions [16,17]. Broader AD literature indicates that B-cell effects can be context-dependent [18,19,20,21], while studies of meningeal immunity identify the meninges and dural sinuses as active neuroimmune interfaces [22,23,24]. These studies provide biological context rather than direct evidence for a skull-marrow mechanism. Thus, the reservoir concept is experimentally supported, whereas its magnitude and clinical significance in human AD remain unresolved.

### 3.1. In Vivo Models for Studying Skull-Bone-Marrow-Derived Immune Cells

A central experimental challenge is to distinguish leukocytes generated in skull bone marrow from cells arriving through the systemic circulation or already resident in the dura. No single model resolves cell origin, route, timing, and function simultaneously. The most informative studies therefore combine spatial labeling or genetic marking with blood and remote-marrow controls, time-resolved imaging, and tissue-level phenotyping. Recent methodological syntheses emphasize that apparent “skull-derived” origin should be interpreted only within the specific strengths and limitations of the model used [25].

#### 3.1.1. Focal Skull-Marrow Labeling and Direct Tracking

The earliest direct approach uses local microinjection of fluorescent tracers into discrete skull-marrow compartments, often with a spectrally distinct tracer injected into tibial marrow as a remote comparator. Hérisson et al. used this strategy in murine stroke and aseptic meningitis and found that labeled neutrophils originating from skull marrow reached the adjacent inflamed CNS surface more readily than tibial-marrow-labeled cells [4]. When coupled to flow cytometry and confocal or intravital microscopy, focal labeling offers strong spatial information and can reveal rapid trafficking through skull channels. Its main limitation is that injection can leak through the same channels being studied and unintentionally label dural cells; only a small dorsal marrow territory is usually tagged, and long-term tracking is limited by dye dilution and turnover [6,25]. Thus, focal labeling is best suited to short time-scale trafficking questions rather than quantitative estimates of the total skull-marrow contribution.

#### 3.1.2. Skull Bone-Flap Transplantation and Local Perturbation

Skull bone-flap transplantation provides a more durable source label. A fluorescently marked calvarial segment from a donor mouse can be transplanted over intact dura in a recipient, allowing donor-marrow cells to be identified after they enter the underlying meninges or CNS. This approach was instrumental in demonstrating a local myeloid contribution to CNS borders [6] and has subsequently been used in glioblastoma to track skull-derived myeloid precursors and tumor-associated neutrophils [26]. The major caveat is surgical: craniotomy and transplantation necessarily interrupt native skull channels, provoke local wound responses, and may alter the marrow niche. Channels can reform, but their regenerated geometry and physiology may not fully reproduce the endogenous state; donor marrow can also be partly replaced by recipient cells over time [25]. Local pharmacological manipulation of intact skull marrow, such as topical or intracalvarial CXCR4 blockade, can complement transplantation by testing whether increased marrow egress changes downstream CNS phenotypes, but such interventions establish functional dependence rather than pure lineage origin [6,26].

#### 3.1.3. Parabiosis and Selective Irradiation/Bone-Marrow Chimeras

Parabiosis joins the circulation of differently labeled mice while leaving each animal’s skull marrow physically in place. Once circulating leukocyte chimerism approaches equilibrium, a persistent excess of host-derived cells in dura or CNS relative to blood supports contribution from a local marrow reservoir. Cugurra et al. used this logic to show that meningeal myeloid populations are less dependent on circulating cells than would be expected if blood were the sole source [6]. The inference is indirect, however, because host enrichment does not visualize a cell leaving skull marrow. Selective irradiation and bone-marrow transplantation provide a complementary perturbational strategy: head irradiation with body shielding, or conversely head shielding with systemic irradiation, can create different donor/host chimerism patterns in cranial versus peripheral marrow [25]. These models can test necessity and source relationships across the entire skull, including basal marrow, but irradiation alters hematopoietic stem/progenitor cells, stromal support, vascular integrity, and inflammatory signaling. Consequently, differences observed after irradiation may reflect niche injury in addition to depletion of skull-derived leukocytes.

#### 3.1.4. Photoconversion and Intravital Multiphoton Imaging

Photoconvertible reporter mice, including Kikume Green-Red (KikGR) systems, permit spatially restricted conversion of fluorescent proteins in the calvarial region and subsequent tracking of converted cells. The method offers high temporal resolution without transplanting bone or systemically irradiating the animal, and it can be combined with flow cytometry of dura, CSF, and brain. Its key limitation is imperfect spatial selectivity: illumination can convert cells in the adjacent dura as well as marrow, so later detection of converted cells does not automatically prove a marrow origin [25]. Intravital two-photon or multiphoton microscopy provides the strongest direct evidence of movement because it can visualize leukocyte behavior within marrow and skull channels in real time. Second- and third-harmonic generation can additionally delineate mineralized bone and channel architecture. Imaging through intact or optically modified skull minimizes disruption but sacrifices depth and resolution; thinning the skull improves optical access at the cost of local injury. Accordingly, live imaging is most convincing when paired with lineage/source labeling and sham-manipulated controls [4,25].

#### 3.1.5. Molecular Profiling as a Necessary Companion to Lineage Models

Lineage-origin experiments answer where cells come from but not what they become. Flow cytometry, single-cell RNA sequencing, spatial imaging, and—where lymphocytes are studied—paired V(D)J/T-cell-receptor sequencing are therefore essential companions. In human glioblastoma, shared T-cell clonotypes between tumor-adjacent cranial marrow and the tumor provide an endogenous molecular “barcode” consistent with functional coupling, although directionality cannot be proven from clonotype overlap alone [27]. For rigorous in vivo studies, the strongest design is triangulation: compare skull and remote marrow, blood, dura, and brain; establish a time course; document channel integrity; and reproduce the inference with at least two mechanistically different source-tracing or perturbation approaches [25]. Principal in vivo models for studying contributions of skull-bone-marrow-derived immune cells to CNS immune surveillance are presented in Table 2.

### 3.2. Pathophysiological Roles in Other Diseased CNS Conditions

The skull-marrow response is not specific to AD. Acute ischemic injury and brain tumors provide complementary disease contexts in which cranial marrow has been interrogated with stronger spatial and functional experiments. These models are important for interpreting AD because they show that the same anatomical reservoir can generate very different immune effects depending on the initiating signal, disease time scale, and recruited lineage.

#### 3.2.1. Ischemic Stroke

Ischemic stroke provided the first direct evidence that skull marrow can serve as a rapid local source of inflammatory myeloid cells. After experimental stroke, Hérisson et al. observed a preferential decline of neutrophils and monocytes in skull marrow relative to tibial marrow and detected a greater contribution of skull-labeled neutrophils to the adjacent inflamed CNS. Live and ex vivo microscopy showed neutrophils traversing microscopic vascular channels through the inner skull cortex toward the dura, including migration against local blood-flow direction [4]. This anatomy creates a short route by which cranial myeloid cells can reach a focal brain injury before a purely systemic marrow response is fully mobilized.

Subsequent work demonstrated that the cranial response to focal ischemia is regionally organized rather than uniform. In a mouse middle-cerebral-artery-occlusion model, Xu et al. used single-cell RNA sequencing, flow cytometry, and cytokine profiling to show stronger hematopoietic activation in skull marrow ipsilateral to the ischemic hemisphere, with prominent neutrophil-biased myeloid responses. Osteopontin signaling contributed to this lateralized activation and meningeal inflammation; experimental inhibition of osteopontin improved neurological outcomes [28]. These data support a pathophysiological model in which the skull marrow acts as a locally tuned sensor–effector compartment after ischemia. The biological effect is likely time- and phenotype-dependent: rapid myeloid recruitment can support debris handling and host defense, whereas excessive neutrophil/monocyte activation and pro-inflammatory signaling may intensify secondary neurovascular injury. Thus, stroke establishes both the feasibility and the potential pathogenic relevance of local cranial hematopoiesis, without implying that the same cell states or kinetics apply to chronic AD.

#### 3.2.2. Brain Tumors

Brain tumors illustrate an even more context-dependent interaction between the CNS and skull marrow. In murine glioblastoma, Lad et al. identified immature neutrophil precursors arising from local skull marrow that enter tumors and differentiate into dendritic-like “hybrid” tumor-associated neutrophils with antigen-processing and MHC-II-dependent T-cell-stimulatory properties. Labeled skull-flap transplantation and targeted skull-marrow ablation supported a local calvarial origin, while intracalvarial CXCR4 blockade with AMD3100 increased marrow egress, enhanced the hybrid neutrophil response, reduced tumor growth, and prolonged survival in the tested models [26]. This study therefore provides causal evidence that skull-derived myeloid cells can exert antitumor functions rather than serving only as inflammatory effectors.

Human glioblastoma data reveal a parallel adaptive-immune dimension. Dobersalske et al. found that tumor-adjacent cranial bone marrow contains active lymphoid populations, including CD8 + effector populations with T-cell-receptor clonotypes shared with the tumor. These cranial-marrow T cells displayed tumor reactivity and a less exhausted functional state than intratumoral counterparts, while 68Ga-Pentixafor PET demonstrated focal CXCR4-associated cranial signals adjacent to glioblastoma [27]. Although shared clonotypes do not prove the direction of trafficking, the study supports a functionally coupled cranioencephalic immune unit in patients.

The tumor can also remodel the skull niche itself. Dubey et al. reported that glioblastoma produces calvarial bone abnormalities in mouse models and patients, increases skull-channel number in mice, and reshapes skull-marrow immunity toward neutrophil expansion with loss of multiple B-cell subsets. Importantly, pharmacological inhibition of bone resorption reduced calvarial abnormalities but worsened progression of mesenchymal-subtype tumors and eliminated the survival benefit of anti-PD-L1 therapy in that model, demonstrating that osteoimmune remodeling of the skull is not merely an epiphenomenon [29]. More recently, Cooper et al. showed in childhood brain-tumor models that tumor-derived signals reaching cranial marrow can drive antigen-presenting hematopoietic stem/progenitor cells toward myelopoiesis and regulatory-T-cell-associated immunotolerance; neutralizing cytokines enriched in tumor-associated CSF normalized hematopoiesis and produced marked tumor regression in the experimental systems [30]. Taken together, brain-tumor studies show that skull marrow can support either antitumor immunity or immunotolerance depending on the tumor and recruited cell state. This bidirectionality is particularly relevant to AD, where the functional sign of skull-derived immune responses should not be assumed from cell origin alone.

## 4. Amyloid in Skull Marrow

Detection of Aβ-related signals in skull marrow provides a direct AD-focused extension of the cranial reservoir framework. The central questions are whether this signal is regionally specific, temporally informative, and biologically active.

The most direct evidence comes from Zhang et al., who reported that Aβ accumulates within the bone marrow of patients with AD and is particularly concentrated in bones surrounding the CNS [14]. Using PET/CT-based amyloid imaging, the authors compared patients with AD dementia and positive brain Aβ-PET scans with non-AD dementia patients who had negative brain Aβ-PET scans. In addition to cerebral Aβ deposition, AD patients showed increased amyloid tracer uptake in the skull and other flat bones, whereas long-bone marrow showed much weaker or absent involvement [14]. Regional skull analyses further indicated higher tracer uptake in frontal, temporal, and occipital skull regions in AD patients compared with non-AD dementia controls, suggesting that skull marrow amyloid signal may show spatial heterogeneity rather than representing a uniform systemic phenomenon [14].

Mouse-model evidence strengthens the human imaging observation. In both 5×FAD and APP/PS1 mice, Zhang et al. detected Aβ accumulation in skull bone marrow before extensive cerebral plaque deposition [14]. In 5×FAD mice, Aβ42 levels in skull marrow supernatant were elevated early and increased with disease progression, whereas femoral marrow did not show the same marked increase; APP/PS1 mice likewise showed skull but not femoral marrow Aβ42 deposition [14]. Immunohistochemistry confirmed skull marrow Aβ accumulation. These findings suggest that skull marrow amyloid involvement can occur relatively early and differs from remote peripheral marrow, but they do not establish that it precedes the initiation of cerebral amyloidosis or contributes causally to disease onset. Possible sources include CSF-derived Aβ, circulating Aβ, and local APP processing within bone-associated tissues [8,9,15,31,32,33].

Experimental findings from Zhang et al. indicate that Aβ-associated marrow changes can alter hematopoiesis in the mouse models studied. Early-stage AD-model mice showed increased hematopoietic stem-cell activity, common lymphoid progenitors, B lymphopoiesis, and expansion of pro-inflammatory age-associated B cells (ABCs) [14]. Aβ promoted B-cell generation and ABC expansion through IL-6 signaling, whereas IL-6 receptor blockade suppressed these responses, delayed cerebral Aβ pathology, and improved cognitive outcomes [14]. ABC-transfer and in vitro experiments further showed that marrow-derived ABCs can increase microglial reactivity and worsen amyloid-related outcomes [14]. Together, these experiments support an immune-amplification mechanism but do not demonstrate that an endogenous skull marrow-to-brain feedback loop operates in sporadic human AD.

Related non-immune skeletal and stromal mechanisms—including Aβ-associated bone remodeling, marrow adipocyte and senescence pathways, and APP-related osteoimmune remodeling—are considered in Section 5 [13,15,33,34].

Whether skull marrow Aβ represents a correlate, consequence, compensatory response, or modifier of cerebral amyloid pathology remains unresolved. Its relatively early appearance in amyloid-based mouse models and its association with IL-6-dependent hematopoietic changes are compatible with an amplifying role, but human directionality has not been established. Alternative explanations include CSF-derived or circulating Aβ, local APP processing, and changes in marrow composition [14]. Thus, the available evidence supports biological activity of skull marrow Aβ in experimental systems, but not a comparable temporal or causal relationship in human AD.

## 5. Osteoimmune Remodeling in AD

Osteoimmune remodeling refers to the reciprocal interaction between bone-forming cells, bone-resorbing cells, stromal elements, vascular structures, marrow adipocytes, and immune populations within the bone marrow niche. In AD, this concept is particularly relevant because the skull bone marrow is not only a hematopoietic compartment but also an active skeletal and immune microenvironment positioned directly adjacent to the meninges and brain. Experimental studies provide evidence that APP/Aβ-related manipulations can affect bone-cell function and skull marrow architecture in selected models. Other features of the marrow microenvironment, including inflammatory cytokine signaling, cellular senescence, marrow adiposity, and vascular remodeling, also occur during normal aging and other systemic conditions and should not be considered AD-specific without direct evidence.

Clinical and experimental observations link AD with reduced bone mineral density, osteoporosis or osteopenia, and fracture risk, although immobility, malnutrition, age, endocrine factors, and falls contribute substantially [35,36,37]. Mechanistically, osteoblasts, osteoclasts, stromal cells, immune cells, and marrow adipocytes express APP-related machinery or respond to inflammatory mediators implicated in AD [38,39,40,41,42,43,44]. This provides a basis for testing whether AD-related molecular pathways directly perturb skeletal and marrow remodeling rather than treating all bone changes as secondary consequences of dementia.

One of the earliest mechanistic links between AD-related genes and bone remodeling came from studies of Swedish mutant APP (APPswe). Xia et al. showed that APPswe suppresses osteoblast differentiation and function, decreases bone formation, enhances osteoclastogenesis, increases marrow adiposity, and produces osteoporotic deficits in mouse models [45]. Importantly, selective expression of APPswe in mature osteoblast-lineage cells was sufficient to reduce osteoblastogenesis and increase osteoclast formation, indicating that APPswe can act directly within the skeletal compartment [45]. These findings indicate that AD-associated APP biology is not confined to neurons or glial cells but can also disrupt the cellular machinery of bone remodeling.

Further work extended this concept from bone loss to brain-relevant inflammation. Pan et al. reported that osteoblast-specific APPswe expression induces endoplasmic reticulum stress-driven senescence in osteoblast-lineage cells and is associated with systemic inflammation, cerebral inflammatory changes, glial activation, and behavioral abnormalities [46]. This study proposed an osteoblastic APPswe-to-brain axis, suggesting that dysfunctional bone-forming cells can generate inflammatory or senescence-associated signals capable of influencing the CNS [46]. For the skull marrow hypothesis, this is highly relevant because osteoblast-lineage cells form part of the calvarial marrow niche and may regulate both hematopoiesis and local vascular remodeling.

The most direct evidence for osteoimmune remodeling of the skull marrow in AD comes from Xiong et al., who identified a skull bone marrow-to-brain axis linking osteoblastic activity, myeloid-cell trafficking, cerebral blood flow, and cognition during AD progression [15]. In APPswe-based mouse models, the authors observed early skull bone marrow changes, including altered marrow cellularity, increased marrow density, and expanded vascular channels connecting the skull marrow to the meninges [15]. In APPswe-based mouse models, these skull marrow alterations occurred alongside changes in skull-derived myeloid-cell trafficking, cerebral blood flow, and cognitive outcomes [15]. However, the available experiments do not establish that osteoimmune remodeling of the skull marrow itself mediates the observed cognitive changes.

In the Xiong et al. model, skull marrow-associated changes varied with age and disease progression [15]. These observations suggest potential stage dependence within that experimental system. However, the available data do not establish that early skull marrow changes are compensatory or that later changes become maladaptive. Such interpretations should remain hypotheses to be tested directly.

Aβ itself may also influence skeletal remodeling. Jung et al. showed that Aβ neuropathology in male mice reduced bone formation and increased osteoclast-associated resorption, including RANKL-related changes [34]. Because the study examined systemic bone rather than skull marrow, it supports an effect of amyloid pathology on bone turnover but does not demonstrate skull-specific remodeling or altered skull–meninges communication.

The marrow microenvironment may further amplify AD-related osteoimmune remodeling through adipocyte and senescence pathways. Bone marrow adipocytes increase with aging and can modify hematopoiesis, inflammation, and amyloid deposition. Kumar et al. reported that senescent bone marrow adipocytes secrete serum amyloid P component, which stabilizes Aβ peptides and promotes skeletal amyloidosis in aged and AD-model mice [33]. Although this work was not restricted to skull marrow, it suggests that age-related marrow remodeling may create a biochemical environment that favors amyloid stabilization and deposition. In the cranial compartment, marrow adiposity, senescence-associated secretory phenotypes, and amyloid-binding proteins may therefore interact with Aβ and inflammatory signals to reshape immune-cell production.

Overall, APP/Aβ-related changes in bone-cell biology, skull marrow architecture, stromal composition, hematopoietic output, and vascular remodeling provide a rationale for investigating skull marrow as an AD-relevant osteoimmune compartment. Because these observations derive from different experimental systems, they should not be interpreted as a single established causal sequence. The principal experimental components are summarized in Table 3.

Taken together, the available findings support a testable model in which skull bone marrow may function as a local cranial amplifier of AD-related signals rather than as an established primary driver of disease. As summarized in Figure 3, Aβ/APP-related and CNS-derived signals may be associated with two partly convergent processes within the marrow compartment: hematopoietic and immune reprogramming and osteoimmune remodeling. These processes may subsequently modify skull marrow immune-cell output and trafficking toward meningeal and CNS-border compartments, with potential consequences for microglial reactivity, neuroinflammation, cerebral blood flow, and amyloid-associated pathology. Importantly, the connections illustrated in Figure 2 represent experimentally supported associations and mechanistic hypotheses of differing evidential strength rather than a demonstrated linear causal pathway in human AD.

## 6. Human Imaging and Translational Evidence

In humans, skull marrow–CNS relationships must be studied primarily through indirect approaches, including MRI, PET/PET-CT, tracer methods, and tissue analysis. These approaches show that cranial drainage, inflammatory, and amyloid-related signals can be measured in vivo, but they do not yet validate skull marrow as an AD biomarker.

The first translational step was the demonstration that CNS-border lymphatic structures can be visualized in humans. Absinta et al. showed that human and nonhuman primate meninges contain lymphatic vessels that can be noninvasively visualized using high-resolution clinical MRI after gadolinium administration [47]. These vessels were enhanced with gadobutrol but not with a blood-pool contrast agent, supporting the interpretation that they represent lymphatic rather than venous structures [47]. This work did not focus on skull bone marrow, but it established that human meningeal lymphatic anatomy can be imaged in vivo. Additional histological work showed that lymphatic vessels are distributed widely in human dura mater, including regions remote from the dural sinuses and with apparent abundance near the skull base [48]. Together, these findings support the translational plausibility of a human cranial border system involved in fluid drainage and immune surveillance.

A more direct link between CSF and skull bone marrow was provided by human intrathecal MRI tracer studies. Ringstad and Eide investigated whether intracranial molecules can move from CSF through the dura into skull bone marrow in patients undergoing work-up for suspected CSF disorders [10]. After intrathecal administration of gadobutrol, tracer enrichment was observed in CSF, parasagittal dura, dural extensions into the skull diploë, and skull bone marrow over a 48-h imaging period [10]. This study provided in vivo evidence that a low-molecular-weight CSF tracer can reach skull marrow regions in humans. Importantly, the authors interpreted this as evidence for molecular trans-dural efflux from intracranial compartments toward the skull bone marrow, suggesting that cranial marrow may participate in human CNS immune surveillance [10].

More recent human tracer-imaging work has extended this idea by examining clinical correlates of skull marrow drainage. Zhou et al. used intrathecal MRI tracer imaging to investigate CSF drainage into skull bone marrow and reported associations between skull marrow tracer dynamics, inflammatory markers, sleep quality, and cognitive performance [12]. Although this work should be interpreted cautiously because it is currently available as an eLife-reviewed preprint, it is highly relevant to AD research because sleep disturbance, impaired CSF clearance, systemic inflammation, and cognitive decline are all central features of the AD risk landscape [12]. The study suggests that skull marrow drainage may be measurable in vivo and may relate to clinically meaningful brain–body variables. However, longitudinal studies in biomarker-defined AD cohorts are still required before skull marrow drainage can be treated as a disease-stage marker.

PET and multimodal molecular studies provide another line of human translational evidence. Kolabas et al. combined mouse transcriptomics, human proteomics, 3D imaging, histology, and TSPO-PET to show that skull marrow has a molecular and functional profile distinct from other bones and that its inflammatory signal can show spatial correspondence with patterns of brain inflammation across neurological disorders [13]. In humans, skull marrow displayed differential immune- and neutrophil-related pathways and a distinct synaptic protein signature [13]. TSPO is not cell-type specific, however, and the observed spatial associations do not establish directionality or functional influence on AD.

AD-specific PET/CT evidence has connected skull bone marrow to amyloid pathology. Zhang et al. reported greater amyloid-related tracer uptake in skull and other flat bones in AD dementia than in amyloid-negative non-AD dementia controls, with higher uptake in frontal, temporal, and occipital skull regions [14]. Because the human component was cross-sectional, it does not establish temporal precedence, and the skull signal could reflect amyloid deposition, marrow composition, inflammation, vascularity, tracer kinetics, bone density, or partial-volume effects. It should therefore be considered an emerging research signal rather than a validated diagnostic marker.

Overall, MRI, TSPO-PET, and amyloid PET/CT demonstrate that skull-associated drainage, inflammatory, and amyloid-related signals can be measured in humans. However, tracer specificity, signal spillover, marrow composition and vascularity, regional anatomical variation, and predominantly cross-sectional study designs limit biological interpretation and currently preclude diagnostic or prognostic use. Longitudinal multimodal studies in biomarker-defined AD cohorts, ideally including remote marrow comparators, are required to establish temporal directionality and determine whether skull marrow imaging provides information beyond established AD biomarkers. The principal human imaging and translational studies are summarized in Table 4.

## 7. Potential Modifiers and Confounders

Interpretation of skull marrow findings in AD requires consideration of biological factors that independently influence hematopoiesis, bone remodeling, inflammation, vascular function, and CNS clearance. Aging is particularly important because it affects AD risk, immune aging, myeloid-biased hematopoiesis, marrow adiposity, bone quality, and meningeal lymphatic function [49,50,53,54]. These age-related processes could alter skull marrow composition or imaging signals independently of AD and should therefore be measured explicitly in future studies.

CSF and lymphatic physiology represent a second group of potential modifiers. Sleep, meningeal lymphatic function, vascular health, and CSF dynamics influence brain clearance and have been associated with cranial tracer behavior [12,49,50,55,56,57]. However, direct evidence that these factors alter AD progression through skull marrow remains lacking. They should therefore be treated primarily as covariates or competing explanations rather than components of an established causal pathway.

Systemic inflammatory state may likewise alter cranial hematopoiesis. Infection and inflammatory signaling can reprogram marrow output, while experimental studies demonstrate that CNS-derived inflammatory signals can reach skull marrow and that inflammatory challenges can recruit skull-derived cells into amyloid-containing brain regions [9,16,58]. Aβ-induced IL-6-dependent B-cell reprogramming provides a more AD-specific example [14], although the roles of other cytokine pathways remain uncertain.

Genetic and sex-related factors may further influence the cranial border environment. APOE4 affects amyloid biology, vascular integrity, lipid metabolism, neuroinflammation, and meningeal lymphatic function, including sex-dependent effects [59,60]. Sex hormones also influence bone remodeling and marrow adiposity. Nevertheless, direct evidence that APOE genotype or sex modifies skull marrow Aβ deposition or hematopoietic responses in human AD is currently unavailable.

Overall, age, bone health, sleep and clearance physiology, systemic inflammation, vascular factors, APOE genotype, sex, and disease stage should currently be treated as potential modifiers or confounders. Their measurement alongside established AD biomarkers will be necessary to determine whether observed skull marrow abnormalities represent AD-related cranial biology or nonspecific effects of aging, inflammation, vascular disease, or skeletal remodeling.

## 8. Experimental Therapeutic Implications and Research Priorities

Therapeutic implications of skull marrow biology remain hypothesis-generating. No skull marrow-directed intervention has been shown to prevent, slow, or treat AD in humans. Current experimental work nevertheless identifies several intervention points for mechanistic study, including Aβ–IL-6-dependent hematopoietic signaling, meningeal lymphatic function, osteoimmune remodeling, skull-derived immune-cell trafficking, and intracalvariosseous drug delivery. These approaches differ substantially in evidential maturity and should not be regarded as components of a validated therapeutic axis.

In the experimental models of Zhang et al., IL-6 receptor blockade reduced marrow B-cell/ABC expansion and delayed cerebral Aβ pathology [14], identifying this pathway as a preclinical intervention point. Translation would require phenotype- and disease-stage-specific targeting because B-cell and IL-6 pathways also support immune surveillance, host defense, hematopoiesis, and tissue repair, and their efficacy and safety in human AD are unknown.

Meningeal lymphatic enhancement improves amyloid-related outcomes in several mouse models [49,50,61,62,63]. Whether these effects are mediated partly through altered skull marrow signaling has not been demonstrated and should be tested directly.

Xiong et al. provide experimental evidence that osteoblastic activity, skull marrow architecture, vascular channels, and AD-relevant outcomes can be linked in an APP-based model [15]. This identifies osteoimmune remodeling as a mechanistic target for further study but does not establish therapeutic efficacy or relevance to sporadic human AD.

Because skull-derived myeloid cells may have context-dependent functions, neither global enhancement nor suppression is currently justified. Future work should identify disease-stage- and phenotype-specific cell states before therapeutic targeting is considered.

A fifth implication is drug delivery. The discovery of skull–meninges microchannels has opened the possibility of using the skull compartment as a route for CNS-targeted therapy. Kang et al. showed that intracalvariosseous administration of donepezil-loaded long-acting microspheres produced sustained brain exposure and improved cognitive performance in mouse models of cognitive impairment [51]. Liu et al. further described intracalvariosseous injection as a CNS drug-delivery approach that uses natural microchannels between skull bone marrow and the dura mater [52]. These findings suggest that the skull marrow–dura interface could be exploited to bypass or complement traditional blood–brain barrier-limited delivery routes. For AD, this could theoretically be relevant for small molecules, biologics, nanoparticles, immunomodulators, or agents designed to target meningeal lymphatics and skull marrow niches.

However, skull-based drug delivery is still far from routine clinical application in AD. Safety questions include infection risk, local bone injury, inflammatory activation, dosing precision, long-term skull marrow effects, and the possibility of altering immune surveillance at the CNS border. Invasive intracalvariosseous strategies may be difficult to justify for chronic neurodegenerative disease unless they offer clear advantages over systemic, intrathecal, intranasal, or focused-ultrasound-assisted approaches. Therefore, skull-based delivery should currently be framed as a preclinical translational opportunity rather than an immediately applicable therapy.

Combination strategies pairing anti-Aβ treatment with lymphatic or marrow-directed interventions remain hypothesis-generating. Their added value has not been demonstrated in patients and will require independent validation of the relevant skull-marrow mechanism.

Skull marrow imaging may eventually serve as an exploratory research endpoint in early-phase translational studies; however, no evidence currently supports its use for patient selection, treatment stratification, or response prediction. Such applications would first require analytical validation, longitudinal association with established AD biomarkers, demonstration of incremental prognostic value, and replication across independent cohorts.

Overall, Aβ–IL-6 hematopoietic signaling, meningeal lymphatic modulation, osteoimmune remodeling, skull-derived immune-cell trafficking, skull-based drug delivery, and exploratory imaging are research directions rather than validated therapeutic strategies in human AD.

## 9. Limitations

The principal limitations arise from the heterogeneity and indirectness of the evidence base. Foundational anatomical studies largely concern non-AD conditions, whereas the strongest AD-specific mechanistic data come from transgenic mouse models and human evidence is predominantly imaging- or association-based. Accordingly, anatomical plausibility, AD-model mechanisms, and human disease relevance should be treated as distinct levels of evidence rather than as a single causal chain.

A major unresolved issue is whether the skull marrow changes discussed in this review represent processes specific to AD or more general responses of cranial hematopoietic and meningeal compartments to CNS inflammation, injury, altered CSF dynamics, or aging. Skull marrow responses have been demonstrated in stroke, meningitis, spinal cord injury, infection, and other neurological conditions, indicating that cranial marrow reactivity is not unique to AD. Accordingly, an association between skull marrow activity and AD does not establish disease specificity. Age-related marrow conversion, systemic inflammation, osteoporosis, vascular disease, altered bone metabolism, peripheral immune activation, and changes in CSF or vascular dynamics may independently or jointly influence skull marrow measurements. Future studies should therefore include neurological, inflammatory, age-matched, and bone-health control groups to determine whether any skull marrow phenotype is specifically associated with AD rather than reflecting a broader CNS-border response. The stroke and brain-tumor literature added in this revision reinforces this concern: the same cranial reservoir can exhibit spatially restricted inflammatory activation, antitumor immune activity, tumor-conditioned myelopoiesis, or osteoimmune remodeling depending on context [4,26,27,28,29,30]. These comparator diseases therefore support the general biological relevance of skull marrow while arguing against disease-nonspecific extrapolation to AD.

A second major limitation is unresolved directionality and stage dependence. Current studies do not establish whether skull marrow abnormalities precede AD pathology, arise secondarily, represent compensatory responses, or amplify established disease. Longitudinal human studies are required to distinguish these possibilities and to determine whether skull marrow effects differ across preclinical, prodromal, and dementia stages.

A further limitation is cellular complexity. Skull marrow contains hematopoietic, stromal, endothelial, osteoblastic, osteoclastic, adipocytic, neural, and vascular components, and current studies often interrogate only one compartment. Consequently, terms such as “skull marrow inflammation,” “B-cell expansion,” or “osteoimmune remodeling” may represent biologically distinct processes that require cell-specific resolution.

Human imaging is limited by tracer specificity, signal spillover, marrow composition and vascularity, regional skull heterogeneity, and non-standardized segmentation approaches. These factors currently limit comparison across studies and preclude clinical interpretation of skull PET/MRI as diagnostic or prognostic biomarkers.

Another unresolved issue is the relative contribution of local cranial routes versus systemic marrow responses. Future studies should compare skull with remote marrow compartments within the same individuals and account for systemic inflammation, aging, vascular disease, and bone health.

Finally, skull marrow measures have not yet been sufficiently integrated with amyloid, tau, neurodegeneration, blood-based biomarkers, or clinical outcomes, and no marrow-directed intervention has established efficacy in AD. These gaps currently limit both mechanistic interpretation and therapeutic translation.

For the skull marrow–meninges–brain framework to gain support beyond anatomical plausibility, future studies should test predictions that distinguish it from alternative explanations. Evidence supporting an AD-relevant local cranial mechanism would include skull marrow alterations that precede or track AD biomarker progression longitudinally, remain associated with disease after adjustment for systemic inflammation and bone-related variables, differ from remote marrow compartments within the same individuals, and predict downstream cerebral or cognitive outcomes independently of established AD biomarkers. Conversely, absence of such regional specificity, temporal relationships, or independent associations would argue that skull marrow abnormalities predominantly reflect systemic, secondary, or nonspecific responses rather than an autonomous contributor to AD pathogenesis.

## 10. Conclusions

Current evidence supports a testable framework in which skull bone marrow is anatomically connected to CNS-border compartments and can respond to CNS-derived signals. AD-specific experimental studies show that Aβ/APP-related changes can alter marrow hematopoiesis, B-cell output, osteoimmune remodeling, and myeloid trafficking. However, most causal evidence derives from transgenic mouse models, whereas human evidence remains predominantly imaging-, tracer-, and association-based. Evidence from ischemic stroke and brain tumors further demonstrates that skull marrow is a dynamic CNS-border immune organ capable of rapid local activation, lineage-specific trafficking, and disease-dependent functional effects [4,26,27,28,29,30].

Accordingly, skull marrow should not yet be considered a causal driver, validated biomarker, or therapeutic target in sporadic human AD. An amplifying role is one plausible interpretation of the available experimental evidence, but alternative explanations—including secondary responses to CNS pathology, systemic inflammation, aging, bone remodeling, and altered CSF or vascular dynamics—remain viable.

The priority is longitudinal, biomarker-defined human research comparing skull with remote marrow compartments and integrating amyloid, tau, neurodegeneration, vascular, inflammatory, and bone-health measures. Such studies are required to determine whether skull marrow alterations precede, accompany, or follow established AD pathology and whether they provide independent diagnostic, prognostic, or therapeutic value.

## Figures and Tables

**Figure 1:**
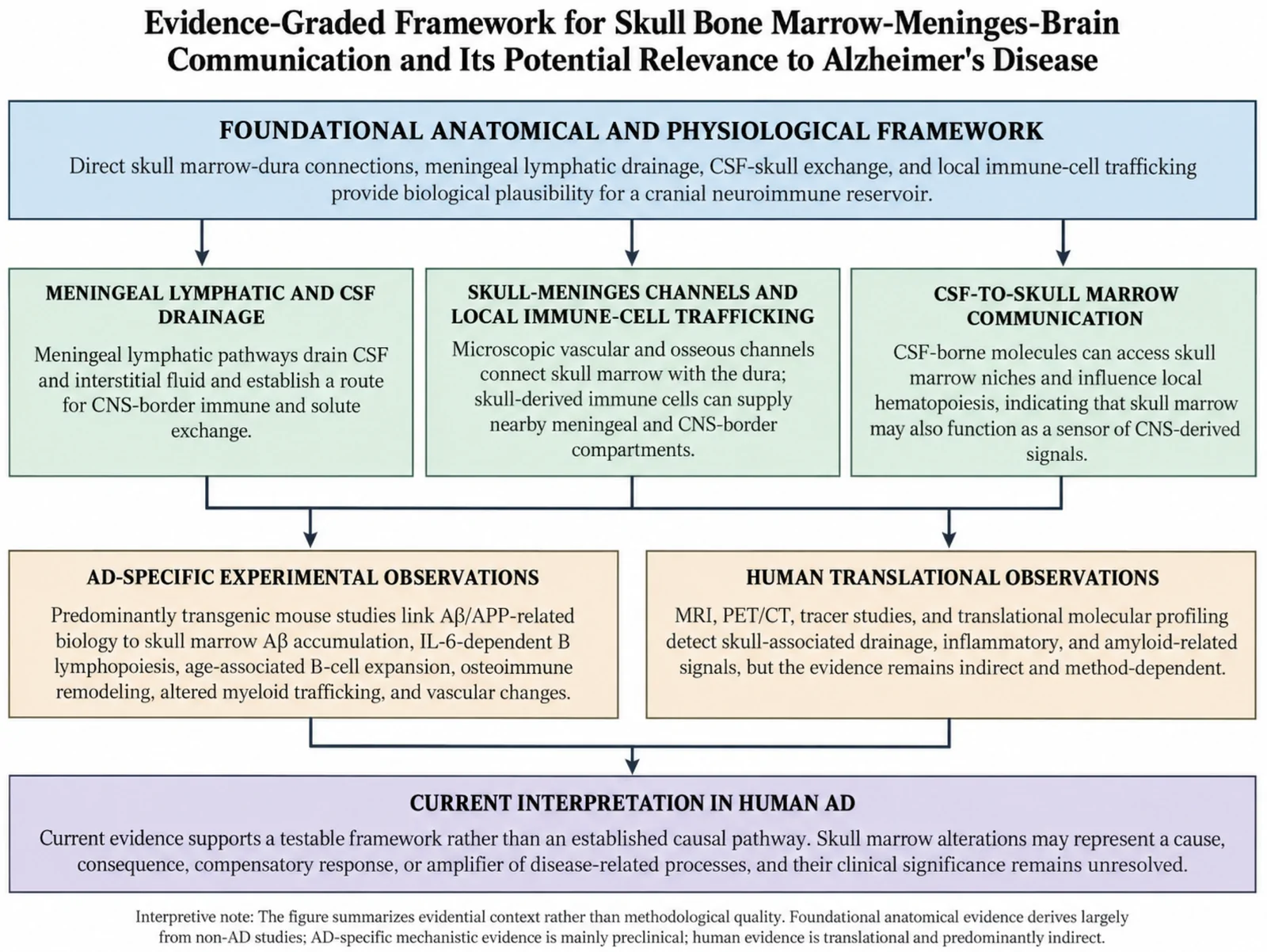
Evidence-graded framework for skull bone marrow–meninges–brain communication and its potential relevance to Alzheimer’s disease. The first bar summarizes the foundational anatomical and physiological framework, including direct skull marrow–dura connections, meningeal lymphatic drainage, CSF-to-skull molecular exchange, and local immune-cell trafficking, which together establish biological plausibility for a cranial neuroimmune reservoir. Arrows from this foundational framework lead to three related evidence domains: meningeal lymphatic and CSF drainage; skull–meninges channels and local immune-cell trafficking; and CSF-to-skull marrow communication. These domains converge on two broader downstream evidence categories: AD-specific experimental observations, derived predominantly from transgenic mouse models, and human translational observations, based mainly on MRI, PET/CT, tracer studies, and translational molecular profiling. The final bar summarizes the current interpretation in human AD, namely that available evidence supports a testable framework rather than an established causal pathway. Skull marrow alterations may represent a cause, consequence, compensatory response, or amplifier of disease-related processes, and their clinical significance remains unresolved. The figure summarizes evidential context and conceptual organization rather than methodological quality or risk of bias.

**Figure 2:**
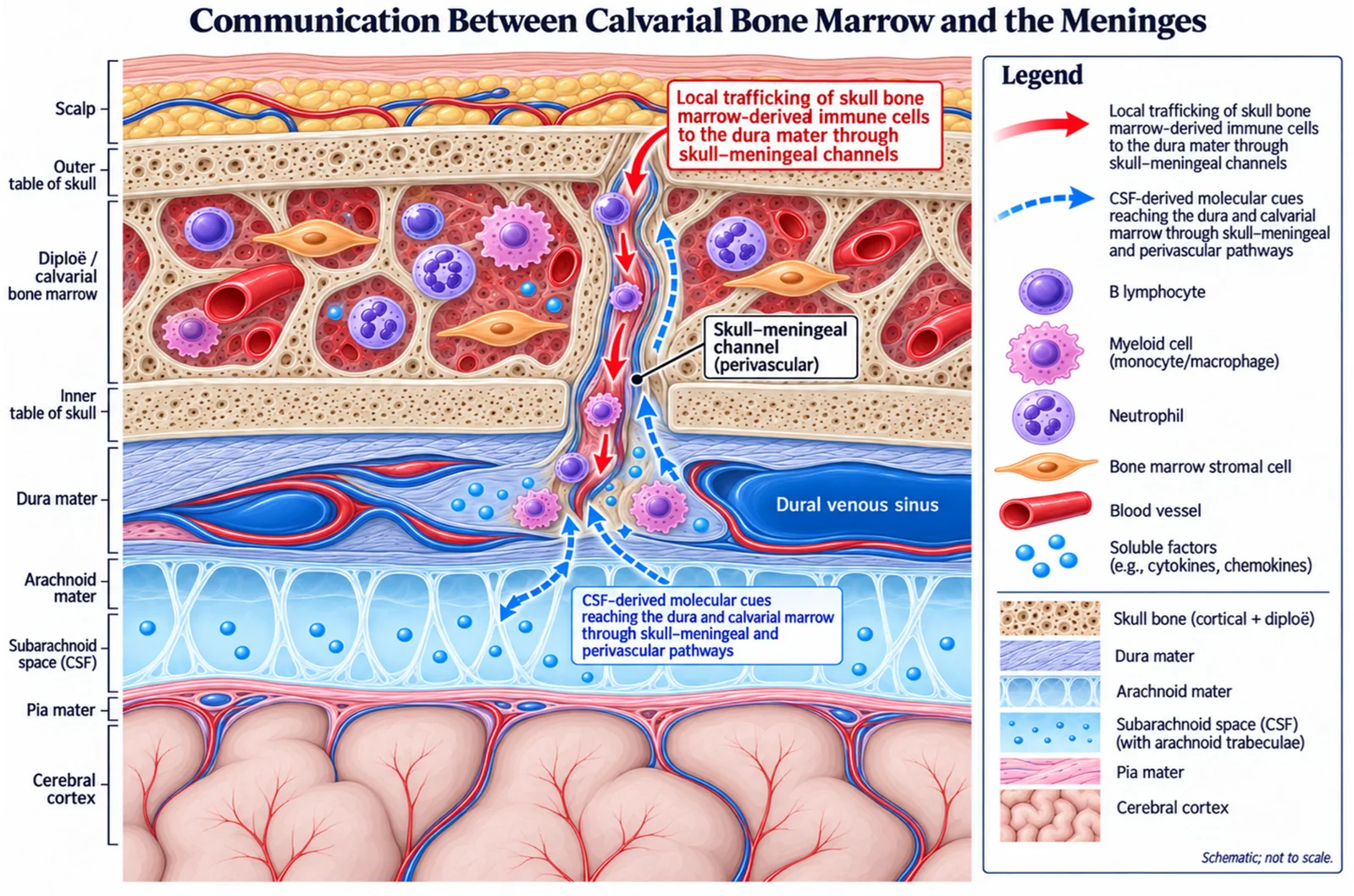
Anatomical schematic (cartoon) of the skull bone marrow–meninges–brain interface. The calvarial marrow lies within the diploic space and is separated from the dura by the inner cortical table, which is traversed by vascular/osseous channels that can support local immune-cell trafficking. The dura contains blood vessels, dural venous sinuses, meningeal immune cells, and lymphatic vessels, whereas the arachnoid encloses the subarachnoid cerebrospinal-fluid (CSF) compartment overlying the pia and superficial cerebral cortex. Red arrows indicate the experimentally supported potential for skull-marrow-derived immune cells to reach the dura and other CNS-border compartments; blue arrows indicate access of CSF-derived molecular cues to skull marrow. The scheme is not to scale and does not imply that every depicted cell type directly enters brain parenchyma. Anatomical relationships are based on the evidence summarized in references [4,5,6,7,8,9,10]. This figure was created using BioRender.

**Figure 3:**
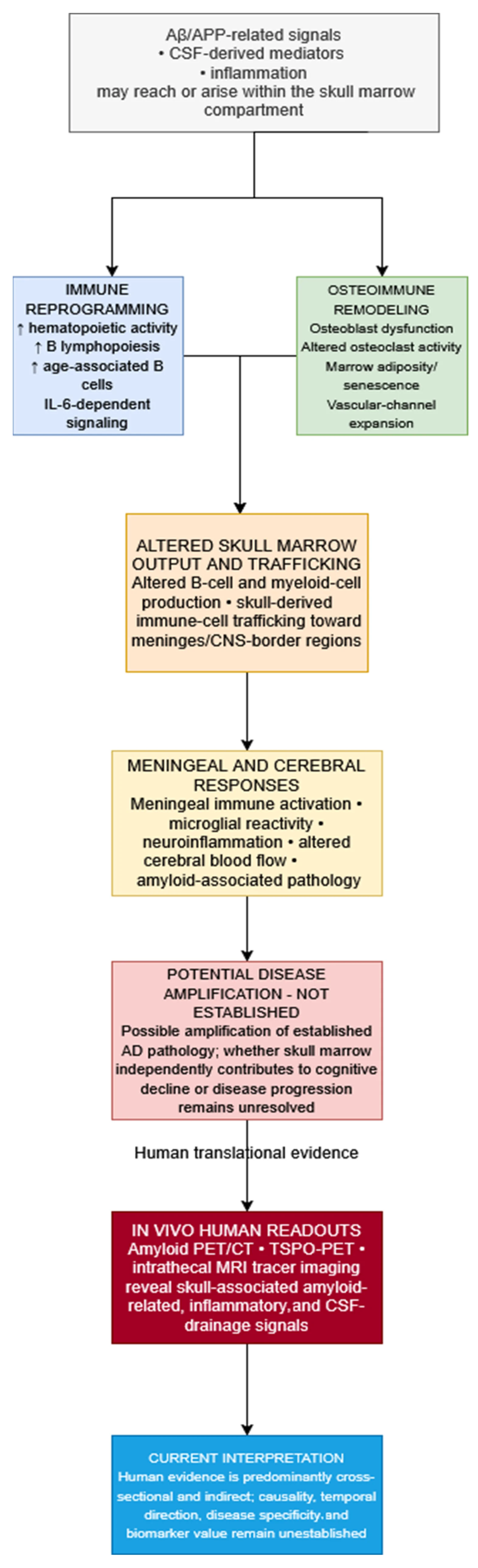
Evidence-based framework of skull bone marrow alterations and their potential relationship with Alzheimer’s disease pathology. Aβ/APP-related and CNS-derived signals may influence the skull bone marrow through two partly convergent processes: hematopoietic and immune reprogramming, including IL-6-dependent B lymphopoiesis and expansion of age-associated B cells, and osteoimmune remodeling, including osteoblast dysfunction, altered osteoclast activity, marrow adiposity and senescence, and expansion of skull–meninges vascular channels. These alterations may modify skull marrow immune-cell production and trafficking toward meningeal and CNS-border compartments, where they could be associated with meningeal immune activation, microglial reactivity, neuroinflammation, altered cerebral blood flow, and amyloid-related pathology. Human PET and MRI studies provide indirect in vivo evidence of skull-associated amyloid-related, inflammatory, and CSF-drainage signals. Arrows indicate experimentally supported or proposed relationships between the summarized processes; they should not be interpreted as demonstrating a continuous causal pathway in human Alzheimer’s disease. Aβ, amyloid-β; AD, Alzheimer’s disease; APP, amyloid precursor protein; CSF, cerebrospinal fluid; IL-6, interleukin-6; MRI, magnetic resonance imaging; PET, positron emission tomography.

**Table 1:** Anatomical and physiological evidence supporting communication between skull bone marrow, the meninges, cerebrospinal fluid, and the brain.

Study	Model/Population	Approach	Key Finding	Interpretation/Evidence Level
A. Meningeal lymphatic and CSF drainage pathways				
Louveau et al., 2015 [1]	Mouse CNS borders and dura mater	Lymphatic-marker staining, microscopy, functional drainage assays	Identified functional meningeal lymphatic vessels along the dural sinuses transporting CSF-derived fluid and immune cells toward deep cervical lymph nodes.	Establishes a pathway for CNS fluid and immune-cell drainage; skull marrow was not examined. Level IV
Aspelund et al., 2015 [2]	Mouse dura, CSF, and brain interstitial-fluid compartments	Lymphatic imaging and tracer-based drainage analysis	Demonstrated dural lymphatic vessels capable of absorbing CSF and brain interstitial fluid and transporting macromolecules toward peripheral lymphatic structures.	Supports CNS-solute drainage through border-associated lymphatic pathways; direct skull marrow involvement was not assessed. Level IV
Ahn et al., 2019 [3]	Mouse skull base and basal meningeal lymphatics	Anatomical and functional lymphatic imaging	Showed specialized meningeal lymphatic vessels at the skull base that constitute an important route for CSF drainage.	Links skull-base anatomy to CSF clearance; does not directly demonstrate skull marrow signaling. Level IV
B. Skull–meninges channels and local immune-cell trafficking				
Hérisson et al., 2018 [4]	Mouse stroke and aseptic meningitis models	Vascular imaging, microscopy, immune-cell tracking	Identified vascular channels connecting skull marrow with the dura and brain surface; skull-derived neutrophils preferentially migrated toward adjacent CNS inflammation.	Establishes local skull marrow immune-cell supply in acute inflammatory models rather than AD. Level IV
Cai et al., 2019 [5]	Whole-body-cleared mice	Panoptic imaging and tissue clearing	Visualized short vascular connections between skull marrow and the meninges, including channels containing immune cells after CNS injury.	Confirms structural continuity between calvarial marrow and meninges; relevance to chronic AD remains inferential. Level IV
C. CSF-to-skull marrow communication				
Mazzitelli et al., 2022 [8]	Mouse skull marrow under homeostatic conditions and after spinal cord injury	CSF tracing, molecular profiling, marrow-cell analysis	Demonstrated direct access of CSF-derived signals to skull marrow niches; injury-associated cues promoted myelopoiesis and myeloid-cell egress toward the meninges.	Supports a local CNS-sensing function of skull marrow; pathological model was spinal cord injury rather than AD. Level IV
Pulous et al., 2022 [9]	Mouse bacterial meningitis model	CSF tracing, infection modeling, hematopoietic analysis	Showed that CSF enters skull marrow through perivascular routes and skull channels and induces cranial hematopoietic responses earlier than remote long-bone marrow.	Supports a local sensor–effector function of cranial marrow; evidence derives from infection rather than neurodegeneration. Level IV
Ringstad and Eide, 2022 [10]	Patients investigated for suspected CSF disorders	Serial intrathecal gadobutrol-enhanced MRI	Detected tracer enrichment in CSF, parasagittal dura, skull diploë, and skull marrow, supporting molecular trans-dural efflux into cranial marrow.	Provides in vivo human evidence for CSF-to-skull marrow exchange; participants were not selected for AD. Level III

**Table 2:** Principal in vivo models for studying contributions of skull-bone-marrow-derived immune cells to CNS immune surveillance.

Model/Approach	What It Establishes	Main Limitation	Representative References
Focal skull-marrow labeling	Short-term, region-specific trafficking and comparison with remote marrow.	Tracer can leak into dura; only a small dorsal marrow region is labeled; dye is not ideal for long-term lineage tracking.	Hérisson et al. [4]; Vinnell et al. [25]
Labeled skull bone-flap transplantation	Durable direct source labeling of cells leaving a transplanted calvarial marrow compartment.	Craniotomy severs native channels and induces wound responses; donor marrow may be repopulated by recipient cells.	Cugurra et al. [6]; Lad et al. [26]
Parabiosis	Tests whether dura/CNS populations are less blood-derived than expected from circulating chimerism.	Source inference is indirect and depends on stable systemic chimerism.	Cugurra et al. [6]; Vinnell et al. [25]
Selective irradiation/bone-marrow chimeras	Tests necessity and broad cranial-versus-peripheral source relationships.	Irradiation alters hematopoietic, stromal and vascular niches and can produce mixed host/donor repopulation.	Vinnell et al. [25]; Lad et al. [26]
Photoconvertible reporters	Provides time-resolved tracking from a defined cranial territory without bone transplantation.	Adjacent dura may also be photoconverted, complicating proof of marrow origin.	Vinnell et al. [25]
Intravital multiphoton imaging	Directly visualizes the route and kinetics of leukocyte movement through skull channels.	Imaging depth is limited; skull manipulation can perturb tissue; lineage/source identity still requires labeling.	Hérisson et al. [4]; Vinnell et al. [25]
Local mobilization or targeted ablation	Tests functional/causal dependence of CNS phenotypes on local skull-marrow activity.	Does not by itself prove lineage route and may affect local stromal/vascular signaling.	Cugurra et al. [6]; Lad et al. [26]

**Table 3:** Experimental evidence for immune and osteoimmune involvement of skull bone marrow in Alzheimer’s disease.

Study	Model/Population	Approach	Key Finding	Interpretation/Evidence Level
A. Hematopoietic and immune-cell mechanisms				
Cugurra et al. [6]	Mouse meninges, skull and vertebral marrow, and CNS parenchyma	Parabiosis, lineage tracing, scRNA-seq, skull transplantation	Demonstrated that skull and vertebral marrow provide local myeloid cells to the meninges and CNS-associated compartments.	Establishes cranial marrow as a local myeloid reservoir; study was not AD-specific. Level IV
Brioschi et al. [7]	Mouse skull marrow and meningeal immune compartments	Single-cell and immunological profiling	Identified heterogeneous meningeal B-cell populations and a lymphopoietic niche supplied partly by skull marrow.	Supports local adaptive immune-cell production at CNS borders; amyloid pathology was not examined. Level IV
Zhang et al. [14]	Patients with AD; 5×FAD and APP/PS1 mice	Amyloid PET/CT, marrow and immune profiling, cell-transfer experiments, IL-6R blockade	Detected Aβ-associated skull marrow changes and showed that Aβ promotes IL-6-dependent B lymphopoiesis and age-associated B-cell expansion, aggravating amyloid-associated outcomes in mouse models.	Strongest direct AD-specific link between skull marrow Aβ and immune reprogramming; human evidence was primarily cross-sectional, whereas causal evidence came from mice. Levels I–II
Yanes et al. [16]	5×FAD mice with chronic Toxoplasma gondii infection	Infection model and immune-cell tracking	Demonstrated recruitment of myeloid cells to amyloid plaques, including skull marrow-derived populations.	Shows that skull-derived immune cells can enter amyloid-containing brain regions; response was infection-driven. Level II
Du et al. [17]	Experimental macrophage-engraftment and amyloid mouse models	Monocyte/macrophage tracing and cellular profiling	Showed that skull marrow-derived and blood-derived macrophages acquire distinct phenotypic and functional properties after brain entry.	Suggests that immune-cell origin may influence function in amyloid pathology; human relevance is unconfirmed. Level II
B. APP/Aβ-associated skull marrow remodeling				
Xiong et al. [15]	APPswe-based AD mouse models	Bone/marrow analysis, vascular assessment, immune-cell tracing, cerebral blood-flow measures	Reported concurrent APP-associated changes in osteoblastic activity, skull marrow cellularity, skull–meninges vascular channels, myeloid trafficking, cerebral blood flow, and cognitive outcomes. Direct mediation of cognitive outcomes by skull marrow osteoimmune remodeling was not established.	Direct experimental evidence for structural, vascular, and immune remodeling of skull marrow; translation to sporadic human AD remains uncertain. Level II
Kumar et al. [33]	Naturally aged and transgenic AD-model mice	Marrow adipocyte, senescence, and amyloid analyses	Found amyloid deposits around senescent marrow adipocytes and showed that adipocyte-derived serum amyloid P stabilizes Aβ and promotes insoluble amyloid formation.	Supports a role of aging-related stromal and adipocyte changes in skeletal amyloid deposition; not specific to skull marrow. Levels II–IV
C. Bone-forming and bone-resorbing cell mechanisms				
Xia et al. [45]	APPswe mouse models and osteoblast-lineage cells	Osteoblast, osteoclast, marrow-adiposity, and bone-density analyses	APPswe suppressed osteoblast differentiation and bone formation, enhanced osteoclastogenesis, increased marrow adiposity, and produced osteoporotic deficits.	Demonstrates direct disruption of skeletal remodeling by AD-associated APP biology; systemic bone rather than skull marrow was examined. Level II
Pan et al. [46]	Osteoblast-specific APPswe mouse model	Senescence, inflammatory, glial, behavioral, and skeletal analyses	Osteoblastic APPswe induced stress-related senescence and was associated with systemic inflammation, cerebral inflammatory changes, glial activation, and behavioral abnormalities.	Supports a bone-to-brain inflammatory pathway involving dysfunctional osteoblast-lineage cells; direct skull marrow-to-brain trafficking was not shown. Level II
Jung et al. [34]	Male mice with Aβ neuropathology	Bone formation, osteoblast, osteoclast, and RANKL-related analyses	Aβ neuropathology induced bone loss by suppressing bone formation and enhancing osteoclast-associated bone resorption.	Supports reciprocal interactions between amyloid pathology and bone turnover; findings were systemic, sex-specific, and not skull-marrow-specific. Level II

**Table 4:** Human imaging, evidence synthesis, and translational evidence concerning the skull marrow–meninges–brain axis in Alzheimer’s disease.

Study	Model/Population	Approach	Key Finding	Interpretation/Evidence Level
A. Human anatomical and imaging evidence				
Absinta et al. [47]	Humans and nonhuman primates	High-resolution contrast-enhanced MRI and histological validation	Demonstrated that meningeal lymphatic vessels can be visualized in vivo and distinguished from adjacent blood vessels.	Establishes feasibility of imaging the human meningeal drainage system; skull marrow and AD were not directly examined. Level III
Ringstad and Eide [10]	Patients investigated for suspected CSF disorders	Serial intrathecal gadobutrol-enhanced MRI	Detected tracer enrichment in CSF, parasagittal dura, skull diploë, and skull marrow over 48 h.	Provides in vivo human evidence for CSF-to-skull molecular exchange; participants were not selected using AD biomarkers. Level III
Zhou et al. [12]	Human intrathecal MRI tracer cohort	Serial MRI tracer imaging with inflammatory, sleep, and cognitive assessments	Reported measurable CSF drainage into skull marrow and associations with inflammation, sleep quality, and cognitive performance.	Suggests clinically relevant correlates of skull marrow drainage; preliminary reviewed preprint and not an AD-specific cohort. Level III
Kolabas et al. [13]	Human neurological cohorts; mouse and human skull tissue	Transcriptomics, proteomics, 3D imaging, histology, and TSPO-PET	Identified molecular specialization of skull marrow and spatial correspondence between skull marrow inflammatory signals and patterns of brain inflammation.	Major translational evidence for skull marrow specialization; TSPO is not cell-type specific and longitudinal AD-specific validation is lacking. Levels III–IV
Zhang et al. [14], human imaging component	AD dementia and amyloid-negative non-AD dementia	Amyloid PET/CT with regional skull analysis	Found greater amyloid-related tracer uptake in skull and other flat bones in AD, including frontal, temporal, and occipital regions.	Demonstrates an AD-associated skull signal in living patients; cross-sectional design and uncertain biological specificity limit interpretation. Level I
B. Evidence synthesis				
Therkelsen et al. [11]	Published studies of brain–meninges–CSF–skull marrow communication	Systematic review of 16 studies	Identified evidence for movement of immune cells, solutes, tracers, bacteria, and therapeutic compounds between intracranial compartments and skull marrow.	Supports the overall communication framework while highlighting major knowledge gaps; most evidence was rodent-based. Levels III–IV
C. Experimental therapeutic and translational evidence				
Da Mesquita et al. [49,50]	Aged mice and AD mouse models, including 5×FAD	Meningeal lymphatic manipulation, imaging, amyloid and microglial analyses, cognitive testing, anti-Aβ immunotherapy	Lymphatic dysfunction aggravated cognitive decline, amyloid pathology, and microglial inflammation and reduced anti-Aβ treatment efficacy; enhancement improved clearance and treatment responses.	Supports meningeal lymphatics as a modifiable component of the cranial system; studies focused on lymphatics rather than skull marrow and remain preclinical. Level II
Kang et al. [51]	Mouse models of cognitive impairment	Intracalvariosseous administration of donepezil-loaded long-acting microspheres	Produced sustained brain exposure to donepezil and improved cognitive performance after skull-based administration.	Proof of therapeutic accessibility of the skull–dura interface; invasive, preclinical, and not established as an AD treatment. Level IV
Liu et al. [52]	Preclinical mouse models	Intracalvariosseous injection through skull–dura microchannels	Demonstrated that natural skull–dura microchannels can be exploited for CNS-targeted drug delivery.	Proof of concept for skull-based CNS delivery; clinical safety, dosing, long-term marrow effects, and comparative efficacy remain unresolved. Level IV

## Data Availability

No new data were created or analyzed in this study. Data sharing is not applicable to this article.

## Supplementary Materials

The following supporting information can be downloaded at: https://www.mdpi.com/article/10.3390/cells15191738/s1, Table S1: Complete database-specific literature-search strategies.
