# Supplementary material for: Skull Bone Marrow as a Cranial Neuroimmune Reservoir in Alzheimer’s Disease: Amyloid, Osteoimmune Remodeling, and Translational Imaging Evidence
Source: Cells. 2026 Sep 23;15(19):1738. doi: 10.3390/cells15191738 (PMC13664314; doi:10.3390/cells15191738)
Supplement: Supplementary file 1 [file cells-15-01738-s001.zip › cells-4566022-supplementary.pdf]

Supplementary table S1. Complete database-specific literature-search strategies.

Database	Search Block	Exact Search Strategy	Search Fields	Time Span	Limits
PubMed/MEDLINE	A. Cranial marrow–CNS-border anatomy and physiology	("skull bone marrow"[Title/Abstract] OR "calvarial bone marrow"[Title/Abstract] OR "cranial bone marrow"[Title/Abstract] OR "skull marrow"[Title/Abstract] OR "calvarial marrow"[Title/Abstract] OR "cranial marrow"[Title/Abstract] OR "skull-meninges"[Title/Abstract] OR "skull meninges"[Title/Abstract] OR "dura-skull channel*"[Title/Abstract] OR "skull-dura channel*"[Title/Abstract] OR "cranial hematopoiesis"[Title/Abstract]) AND (mening*[Title/Abstract] OR dura*[Title/Abstract] OR brain[Title/Abstract] OR CNS[Title/Abstract] OR "central nervous system"[Title/Abstract] OR CSF[Title/Abstract] OR "cerebrospinal fluid"[Title/Abstract] OR lymphatic*[Title/Abstract] OR immune*[Title/Abstract] OR hematopoi*[Title/Abstract] OR myeloid[Title/Abstract] OR lymphoid[Title/Abstract])	Title/Abstract	Database inception–11 June 2026	English; no publication-date restriction
PubMed/MEDLINE	B. Alzheimer's disease/amyloid and cranial or bone marrow	("skull bone marrow"[Title/Abstract] OR "calvarial bone marrow"[Title/Abstract] OR "cranial bone marrow"[Title/Abstract] OR "skull marrow"[Title/Abstract] OR "calvarial marrow"[Title/Abstract] OR "cranial marrow"[Title/Abstract] OR "bone marrow"[Title/Abstract]) AND ("Alzheimer Disease"[MeSH Terms] OR Alzheimer*[Title/Abstract] OR	MeSH + Title/Abstract	Database inception–11 June 2026	English; no publication-date restriction

		amyloid*[Title/Abstract] OR "amyloid beta"[Title/Abstract] OR "amyloid-β"[Title/Abstract] OR "A beta"[Title/Abstract] OR Aβ[Title/Abstract] OR APP[Title/Abstract] OR "amyloid precursor protein"[Title/Abstract]) AND (brain[Title/Abstract] OR CNS[Title/Abstract] OR mening*[Title/Abstract] OR dura*[Title/Abstract] OR CSF[Title/Abstract] OR "cerebrospinal fluid"[Title/Abstract] OR hematopoi*[Title/Abstract] OR immune*[Title/Abstract] OR myeloid[Title/Abstract] OR lymphoid[Title/Abstract] OR osteoblast*[Title/Abstract] OR osteoclast*[Title/Abstract] OR osteoimmun*[Title/Abstract] OR neuroinflamm*[Title/Abstract])			
Scopus	A. Cranial marrow–CNS-border anatomy and physiology	TITLE-ABS-KEY("skull bone marrow" OR "calvarial bone marrow" OR "cranial bone marrow" OR "skull marrow" OR "calvarial marrow" OR "cranial marrow" OR "skull-meninges" OR "skull meninges" OR "dura-skull channel*" OR "skull-dura channel*" OR "cranial hematopoiesis") AND TITLE-ABS-KEY(mening* OR dura* OR brain OR CNS OR "central nervous system" OR CSF OR "cerebrospinal fluid" OR lymphatic* OR immune* OR hematopoi* OR myeloid OR lymphoid)	TITLE-ABS-KEY	Database inception–11 June 2026	English; no publication-date restriction
Scopus	B. Alzheimer's disease/amyloid and cranial or bone marrow	TITLE-ABS-KEY("skull bone marrow" OR "calvarial bone marrow" OR "cranial bone marrow" OR "skull marrow" OR "calvarial marrow" OR "cranial marrow" OR "bone marrow") AND TITLE-ABS-KEY(Alzheimer* OR	TITLE-ABS-KEY	Database inception–11 June 2026	English; no publication-date restriction

		amyloid* OR "amyloid beta" OR "amyloid-β" OR "A beta" OR Aβ OR APP OR "amyloid precursor protein") AND TITLE-ABS-KEY(brain OR CNS OR mening* OR dura* OR CSF OR "cerebrospinal fluid" OR hematopoi* OR immune* OR myeloid OR lymphoid OR osteoblast* OR osteoclast* OR osteoimmun* OR neuroinflamm*)			
Web of Science Core Collection	A. Cranial marrow–CNS-border anatomy and physiology	TS = ("skull bone marrow" OR "calvarial bone marrow" OR "cranial bone marrow" OR "skull marrow" OR "calvarial marrow" OR "cranial marrow" OR "skull-meninges" OR "skull meninges" OR "dura-skull channel*" OR "skull-dura channel*" OR "cranial hematopoiesis") AND TS = (mening* OR dura* OR brain OR CNS OR "central nervous system" OR CSF OR "cerebrospinal fluid" OR lymphatic* OR immune* OR hematopoi* OR myeloid OR lymphoid)	Topic (TS: title, abstract, author keywords, Keywords Plus)	Database inception–11 June 2026	English; no publication-date restriction
Web of Science Core Collection	B. Alzheimer's disease/amyloid and cranial or bone marrow	TS = ("skull bone marrow" OR "calvarial bone marrow" OR "cranial bone marrow" OR "skull marrow" OR "calvarial marrow" OR "cranial marrow" OR "bone marrow") AND TS = (Alzheimer* OR amyloid* OR "amyloid beta" OR "amyloid-β" OR "A beta" OR Aβ OR APP OR "amyloid precursor protein") AND TS = (brain OR CNS OR mening* OR dura* OR CSF OR "cerebrospinal fluid" OR hematopoi* OR immune* OR myeloid OR lymphoid OR osteoblast* OR osteoclast* OR osteoimmun* OR neuroinflamm*)	Topic (TS)	Database inception–11 June 2026	English; no publication-date restriction

Abbreviations: AD, Alzheimer's disease; APP, amyloid precursor protein; CNS, central nervous system; CSF, cerebrospinal fluid; MeSH, Medical Subject Headings; TITLE-ABS-KEY, title, abstract, and keywords; TS, Web of Science Topic field. The asterisk (*) denotes truncation used to retrieve variant word endings. The primary database searches covered database inception through 11 June 2026. The revision-focused supplementary search was conducted separately through 18 September 2026. Only English-language publications were considered, and no publication-date restriction was applied.
